# 4060 A Telehealth Approach to Improving Healthcare to Rural and Underserved Populations

**DOI:** 10.1017/cts.2020.199

**Published:** 2020-07-29

**Authors:** Erica Francis, Jennifer Kraschnewski, Ruth Hogentogler, Kimberly Buckner, Jackie Sabol, Kara Bowers

**Affiliations:** 1 Penn State Clinical and Translational Science Institute

## Abstract

OBJECTIVES/GOALS: Project Extension for Community Health Outcomes (ECHO), a telehealth model, was launched at Penn State University in 2018 to connect specialists with community providers to provide education on best clinical practices. We aim to describe clinical topics covered and relevant provider level outcomes. METHODS/STUDY POPULATION: The heart of the ECHO model is a hub-and-spoke knowledge-sharing system. The ECHO model has four core principles: 1) use technology to leverage scarce resources; 2) share best practices to reduce disparities; 3) employ case-based learning to master complexity; 4) monitor outcomes to ensure benefit. Unlike telemedicine, where outside specialists assume the care of the patient, Project ECHO is a guided learning community aimed at practice improvement: providers receive mentoring and feedback on de-identified patient cases, strengthen their skillset, and retain responsibility for their patients. RESULTS/ANTICIPATED RESULTS: Clinical topics launched include Medication Assisted Treatment for Opioid Use Disorder, Ehlers Danlos Syndrome, Polyneuropathy, and Dementia. In addition, we launched a nutrition-focused ECHO with Boy Scout summer camp leaders in 26 states, reaching 107,347 scouts. Over the past year we have reached 118 clinicians in 62 clinics within 19 counties in Pennsylvania, providing a total of 268 CME hours. These providers have treated 2,294 patients and reported increased knowledge (94%), decreased sense of professional isolation (86%), and improvement in ability to provide patient care (92%) following completion of an ECHO series. DISCUSSION/SIGNIFICANCE OF IMPACT: Project ECHO is a powerful telehealth model providing mentorship and education to clinicians, encouraging them to treat more complex cases in their primary care clinics. As a result, patients receive higher quality care when they need it, and close to home, particularly important in rural areas.

